# Draft Genome Sequence of *Helicobacter fennelliae* Strain MRY12-0050, Isolated from a Bacteremia Patient

**DOI:** 10.1128/genomeA.00512-13

**Published:** 2013-08-08

**Authors:** Emiko Rimbara, Mari Matsui, Shigetarou Mori, Satowa Suzuki, Masato Suzuki, Hyun Kim, Tsuyoshi Sekizuka, Makoto Kuroda, Keigo Shibayama

**Affiliations:** Department of Bacteriology II, National Institute of Infectious Diseases, Musashimurayama-shi, Tokyo, Japana; Laboratory of Bacterial Genomics, Pathogen Genomics Center, National Institute of Infectious Diseases, Shinjuku-ku, Tokyo, Japanb

## Abstract

*Helicobacter fennelliae*, a human enterohepatic pathogen, causes bacteremia and colitis. We isolated *H. fennelliae* strain MRY12-0050 from a female patient; this strain was isolated from 2 other patients from the same hospital during the same period, suggesting human-to-human transmission. This is the first report of an *H. fennelliae* genome sequence.

## GENOME ANNOUNCEMENT

*Helicobacter* species are Gram-negative, spiral bacteria that are categorized into 2 groups, (i) gastric helicobacters and (ii) enterohepatic helicobacters. *Helicobacter pylori* is a major gastric *Helicobacter*, and the genome sequences of several gastric *Helicobacter* strains have been analyzed so far. On the other hand, analyses of genomic sequences for enterohepatic helicobacters have been limited. *H. fennelliae* is an enterohepatic *Helicobacter* that causes bacteremia mainly in immunocompromised hosts (1–3). In this study, *H. fennelliae* strain MRY12-0050 was isolated from the blood of a female patient with non-Hodgkin lymphoma. At first, the strain was misidentified as *H. cinaedi* because the strains have similar morphologies. It was later identified as *H. fennelliae* by sequencing of both the 16S rRNA and 23S rRNA genes. *H. fennelliae* was isolated from 2 other patients from the same hospital ward. Pulsed-field gel electrophoresis (PGFE) of 3 isolates showed the same PFGE pattern between isolates, suggesting human-to-human transmission (4). Since genome-sequencing data for *H. fennelliae* have not yet been reported, we herein describe a draft genome sequence for *H. fennelliae* MRY 12-0050, which causes nosocomial infection.

*H. fennelliae* was cultured in brucella agar (Becton Dickinson, NJ) containing 5% horse blood under microaerobic conditions with hydrogen, which was provided by the gas replacement method by using an anaerobic gas mixture (i.e., H_2_, 10%; CO_2_, 10%; and N_2_, 80%). Genomic DNA was prepared, and a Roche 454 Life Sciences genome sequencer junior system was used to generate DNA sequences at 32× coverage. The same genomic DNA was analyzed by the Illumina MiSeq system paired-end sequences, and the resulting sequence represents 127× coverage. Sequences obtained by using the Roche 454 were assembled using Newbler Assembler v2.7, whereas sequences obtained by using the Illumina MiSeq were assembled with Geneious. The assembled sequences were merged by using Geneious to generate 49 contigs. The contig *N*_50_ was approximately 157 kb in length, and the largest contig assembled was approximately 300 kb. Finally, the DNA sequences were annotated by using the RAST (Rapid Annotation using Subsystem Technology) server (5).

The whole genome is 2.15 Mb in size, has a G+C content of 37.9%, and contains 2,507 genes (2,467 protein-coding genes and 40 structural RNAs). RAST annotation showed that *H. cinaedi* strain CCUG 18818 (score 502), *H. hepaticus* strain ATCC 51449 (score 456), and *H. canadensis* strain MIT 98-5491 (score 444) are the closest neighbors of *H. fennelliae* MRY12-0050. Although *H. cinaedi* and *H. hepaticus* have been known to express cytolethal distending toxin (CDT), which causes DNA damage to target cells, a CDT cluster was not identified in *H. fennelliae* MRY12-0050. Because both *H. cinaedi* and *H. fennelliae* are likely to cause a nosocomial infection and bacteremia in humans, further analyses are needed to elucidate the pathogenic factor in *H. fennelliae*.

### Nucleotide sequence accession numbers.

This whole-genome shotgun project has been deposited at DDBJ/EMBL/GenBank under the accession no. BASD00000000. The version described in this paper is the first version, BASD01000000.

## References

[1] Burnens AP Stanley J Schaad UB Nicolet J 1993 Novel campylobacter-like organism resembling *Helicobacter fennelliae* isolated from a boy with gastroenteritis and from dogs. J. Clin. Microbiol. 31:1916–1917 8349774 10.1128/jcm.31.7.1916-1917.1993 PMC265659

[2] Hsueh PR Teng LJ Hung CC Chen YC Yang PC Ho SW Luh KT 1999 Septic shock due to *Helicobacter fennelliae* in a non-human immunodeficiency virus-infected heterosexual patient. J. Clin. Microbiol. 37:2084–2086 10325388 10.1128/jcm.37.6.2084-2086.1999 PMC85042

[3] Smuts HE Lastovica AJ 2011 Molecular characterization of the 16S rRNA gene of *Helicobacter fennelliae* Isolated from stools and blood cultures from paediatric patients in South Africa. J. Pathog. 10.4061/2011/217376 PMC3335488 22567323

[4] Rimbara E Mori S Kim H Matsui M Suzuki S Takahashi S Yamamoto S Mukai M Shibayama K 2013 *Helicobacter cinaedi* and *Helicobacter fennelliae* transmission in a hospital from 2008 to 2012. J. Clin. Microbiol. 51:2439–2442 23658263 10.1128/JCM.01035-13 PMC3697726

[5] Aziz RK Bartels D Best AA DeJongh M Disz T Edwards RA Formsma K Gerdes S Glass EM Kubal M Meyer F Olsen GJ Olson R Osterman AL Overbeek RA McNeil LK Paarmann D Paczian T Parrello B Pusch GD Reich C Stevens R Vassieva O Vonstein V Wilke A Zagnitko O 2008 The RAST server: rapid annotations using subsystems technology. BMC Genomics 9:75. 10.1186/1471-2164-9-75 18261238 PMC2265698

