# Correction for Rimbara et al., Draft Genome Sequence of *Helicobacter fennelliae* Strain MRY12-0050, Isolated from a Bacteremia Patient

**DOI:** 10.1128/genomeA.00634-16

**Published:** 2016-07-21

**Authors:** Emiko Rimbara, Mari Matsui, Shigetarou Mori, Satowa Suzuki, Masato Suzuki, Hyun Kim, Tsuyoshi Sekizuka, Makoto Kuroda, Keigo Shibayama

**Affiliations:** a Department of Bacteriology II, National Institute of Infectious Diseases, Musashimurayama-shi, Tokyo, Japan; b Laboratory of Bacterial Genomics, Pathogen Genomics Center, National Institute of Infectious Diseases, Shinjuku-ku, Tokyo, Japan

## AUTHOR CORRECTION

Volume 1, no. 4, e00512-13, 2013. After publication, we detected the presence of a putative cytolethal distending toxin (CDT) gene cluster in *Helicobacter fennelliae* MRY12-0050. In the last paragraph before the accession numbers, the third sentence should read as follows: “*H. cinaedi* and *H. hepaticus* have been reported to express cytolethal distending toxin (CDT), which causes DNA damage to target cells, and a putative CDT gene cluster was also identified in *H. fennelliae* MRY12-0050 (with the gene product sharing 31%, 36%, and 32% amino acid sequence identity with CdtA, CdtB, and CdtC, respectively, of *H. cinaedi* strain CCUG 18818).”

